# Supplementary material for: The CBP KIX domain regulates long-term memory and circadian activity
Source: BMC Biol. 2020 Oct 29;18:155. doi: 10.1186/s12915-020-00886-1 (PMC7597000; doi:10.1186/s12915-020-00886-1)
Supplement: Supplementary file 1 — Additional file 1: Fig. S1. CBPKIX/KIX mice show normal learning, reduced swim speed and impaired long-term memory. a CBPKIX/KIX mice show similar performance during trial 1 across training days 1–4 but shows significantly higher escape latency on day 5. 2-Way ANOVA: significant main effect of training days: F (3.318, 99.55) = 4.538, p = 0.0038, significant main effect of genotype: F (1, 30) = 9.507, p = 0.0044. Sidak’s multiple comparisons test, comparing CBPKIX/KIX vs WT on Day 5: adjusted p = 0.0120. b CBPKIX/KIX mice showed lower swim speed during the training days (acquisition). Significant main effect of genotype: F (1, 30) = 15.33, p = 0.0005. Sidak’s multiple comparisons test revealed significant decrease in swim speed (CBPKIX/KIX vs WT mice) on day 4 (p = 0.0067) and day 5 (p = 0.0417). c Male and female CBPKIX/KIX mice showed similar performances during the 1 h probe test (short-term memory). d Male and female CBPKIX/KIX mice showed similar performances in the 24 h probes test (long-term memory). Mixed-effects analysis: Significant main effect of Genotype F (1, 12) = 11.36, p = 0.0056. No significant main effect of sex F (1, 12) = 0.3330, p = 0.5746. Fig. S2. Learning-induced CREB phosphorylation at S133 is decreased in CBPKIX/KIX mice. (a) Scheme of the experiment. (b) CREB phosphorylation at S133 is significantly increased after MWM training ([Unpaired t-test: t(11) = 2.407, p = 0.0348, WT HC Vs WT MWM], while no such enhancement were observed in CBPKIX/KIX mice [Unpaired t-test: t(7) = 0.7450, p = 0.4805]. (c) Western blot showing CREB and p-CREB expression. Fig. S3. RRHO analysis. a Rank-rank hypergeometric overlap (RRHO) analysis comparing differential expression results between the effect of the KIX genotype after learning (X-axis) to the effect of learning (Y-axis). Although a threshold of FDR < = 0.05 corresponds to the top 158 and 135 genes for the X and Y axis, respectively, we observe maximum hypergeometric enrichment between the two lists when [file 12915_2020_886_MOESM1_ESM.docx]

## Supporting Figures for “The CBP KIX domain regulates long-term memory and circadian activity”

Snehajyoti Chatterjee^1,2,3#^, Christopher C. Angelakos^4,5#^, Ethan Bahl^6,7^, Joshua D. Hawk ^4,5^, Marie E. Gaine^3^, Shane G. Poplawski^4,5,8^, Anne Schneider-Anthony^1,2^, Manish Yadav^3^, Giulia S. Porcari^5^, Jean-Christophe Cassel^1^, K. Peter Giese^9^, Jacob J. Michaelson^6,10,11,12^, Lisa C. Lyons^3,13^, Anne-Laurence Boutillier^1,2^* and Ted Abel^3^*

^1^ Laboratoire de Neuroscience Cognitives et Adaptatives (LNCA), Université de Strasbourg, Strasbourg, France

^2^ LNCA, CNRS UMR 7364, Strasbourg, France

^3^ Department of Neuroscience and Pharmacology, Iowa Neuroscience Institute, Carver College of Medicine, University of Iowa, Iowa City, Iowa, United States

^4^ Neuroscience Graduate Group, University of Pennsylvania, Philadelphia

^5^ Department of Biology, University of Pennsylvania, Philadelphia, PA

^6^ Department of Psychiatry, Carver College of Medicine, University of Iowa, Iowa City, Iowa

^7^ Interdisciplinary Graduate Program in Genetics, University of Iowa, Iowa City, Iowa

^8^ Pharmacology Graduate Group, University of Pennsylvania, Philadelphia

^9^ Department of Basic and Clinical Neuroscience, King's College London, London, UK

^10^ Department of Biomedical Engineering, College of Engineering, University of Iowa, Iowa City, Iowa

^11^ Department of Communication Sciences and Disorders, College of Liberal Arts and Sciences, University of Iowa, Iowa City, Iowa

^12^ Iowa Institute of Human Genetics, University of Iowa, Iowa City, Iowa

^13^ Program in Neuroscience, Department of Biological Science, Florida State University, Tallahassee, Florida

^#^ Authors contributed equally

* Corresponding authors: ted-abel@uiowa.edu, laurette@unistra.fr

**Figure S1.**

**
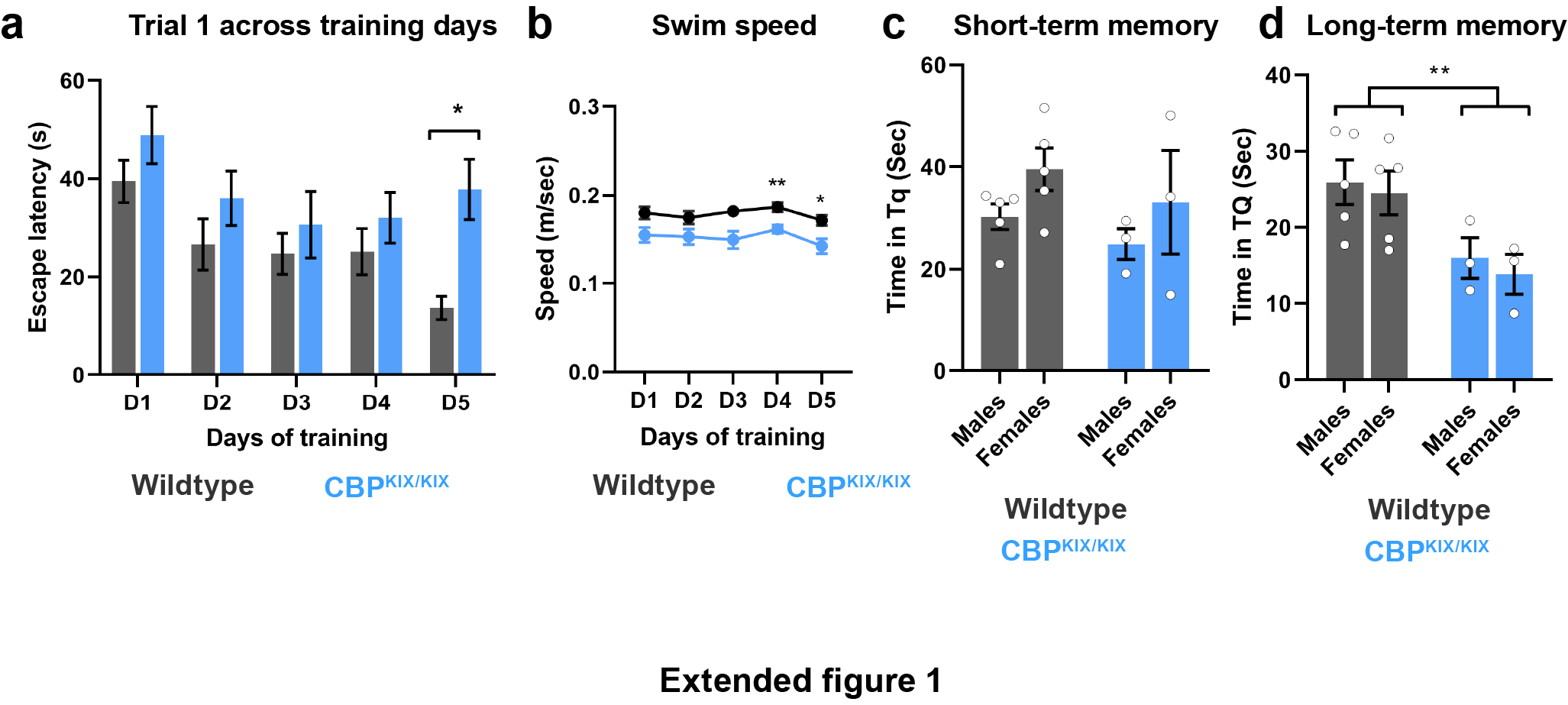
**

**Figure S2.**

**Figure S3.**


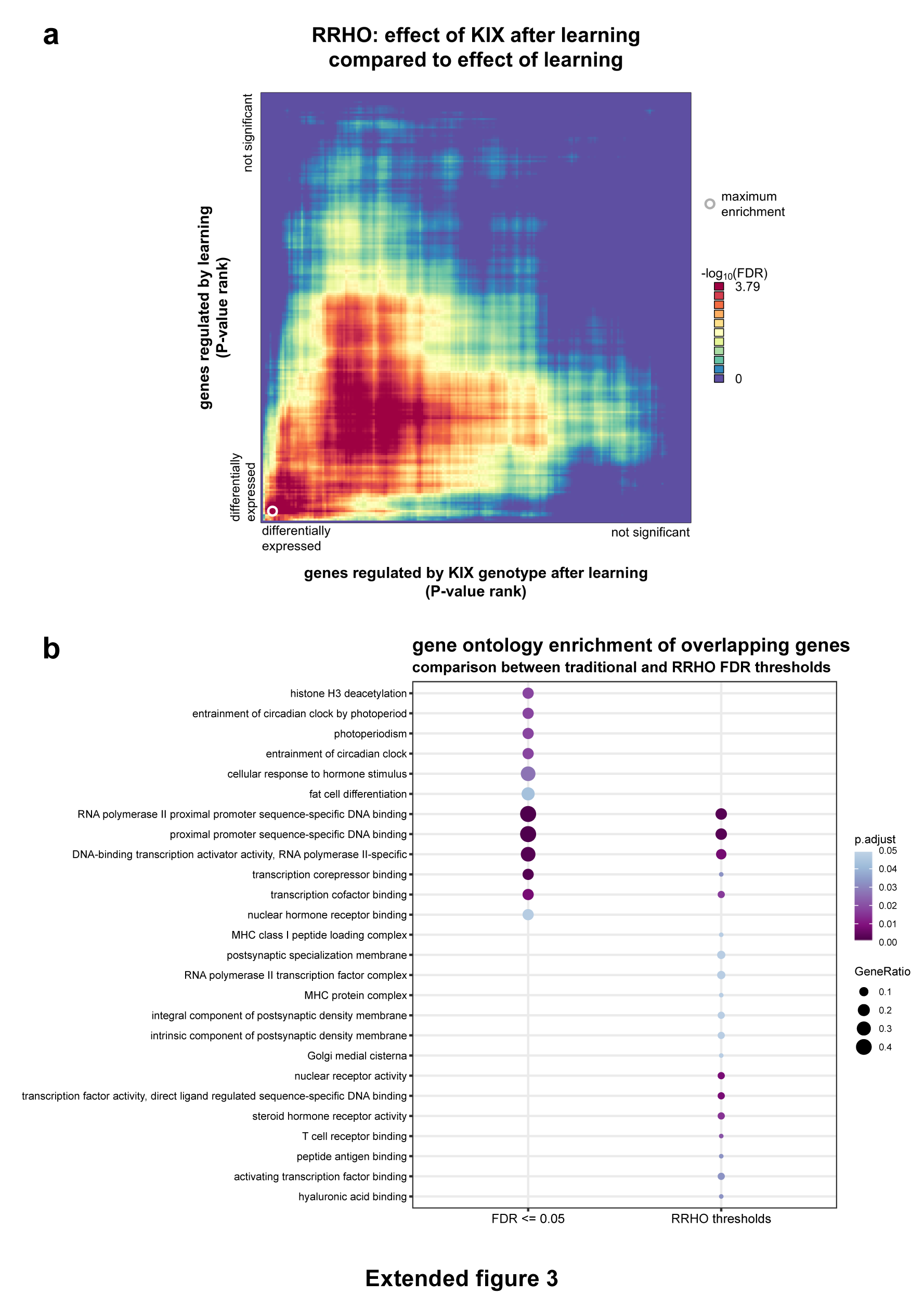


**Figure S4.**

**Figure S5.**


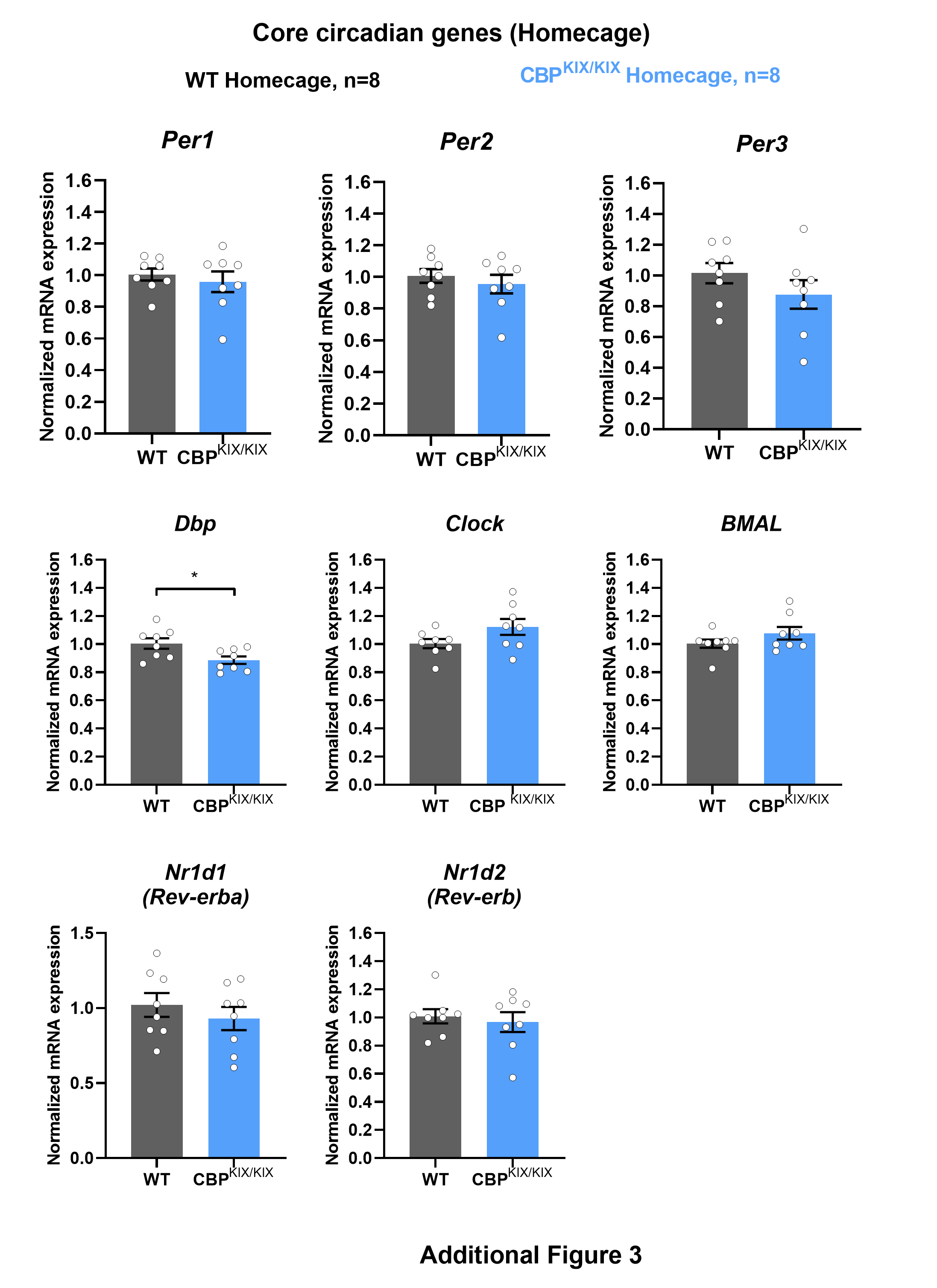


**Figure S6.**

**
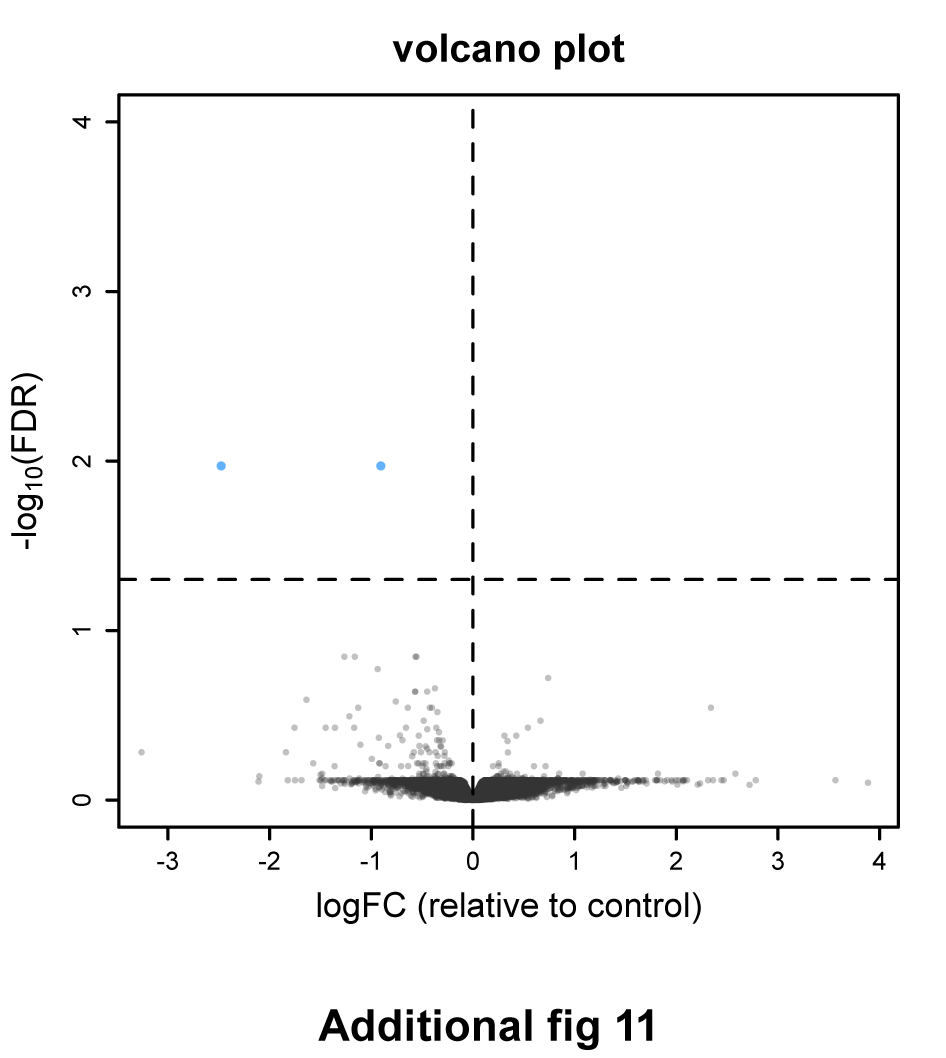
**

**Figure S7.**

**Figure S8.**


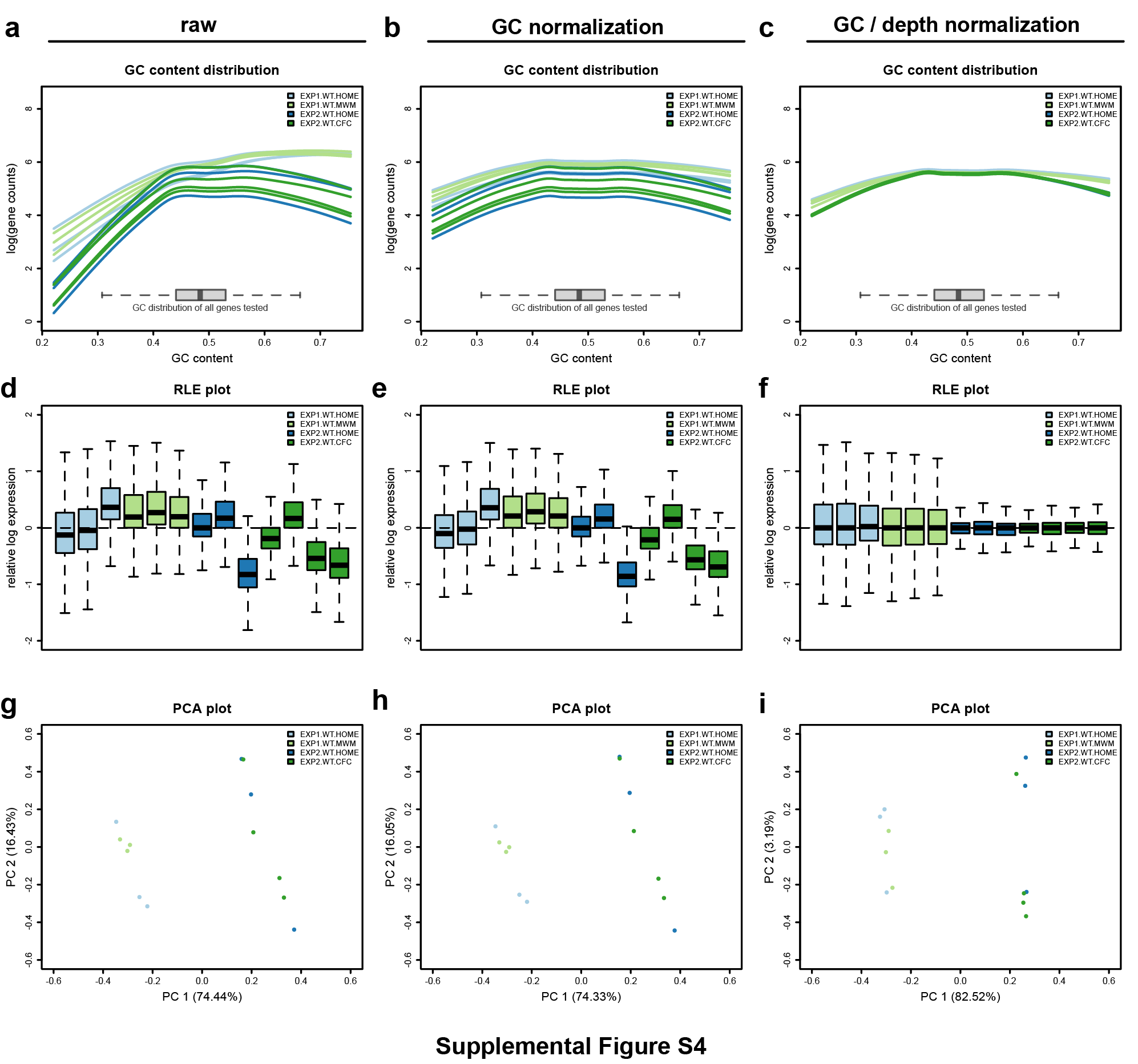


**Figure S9.**

**
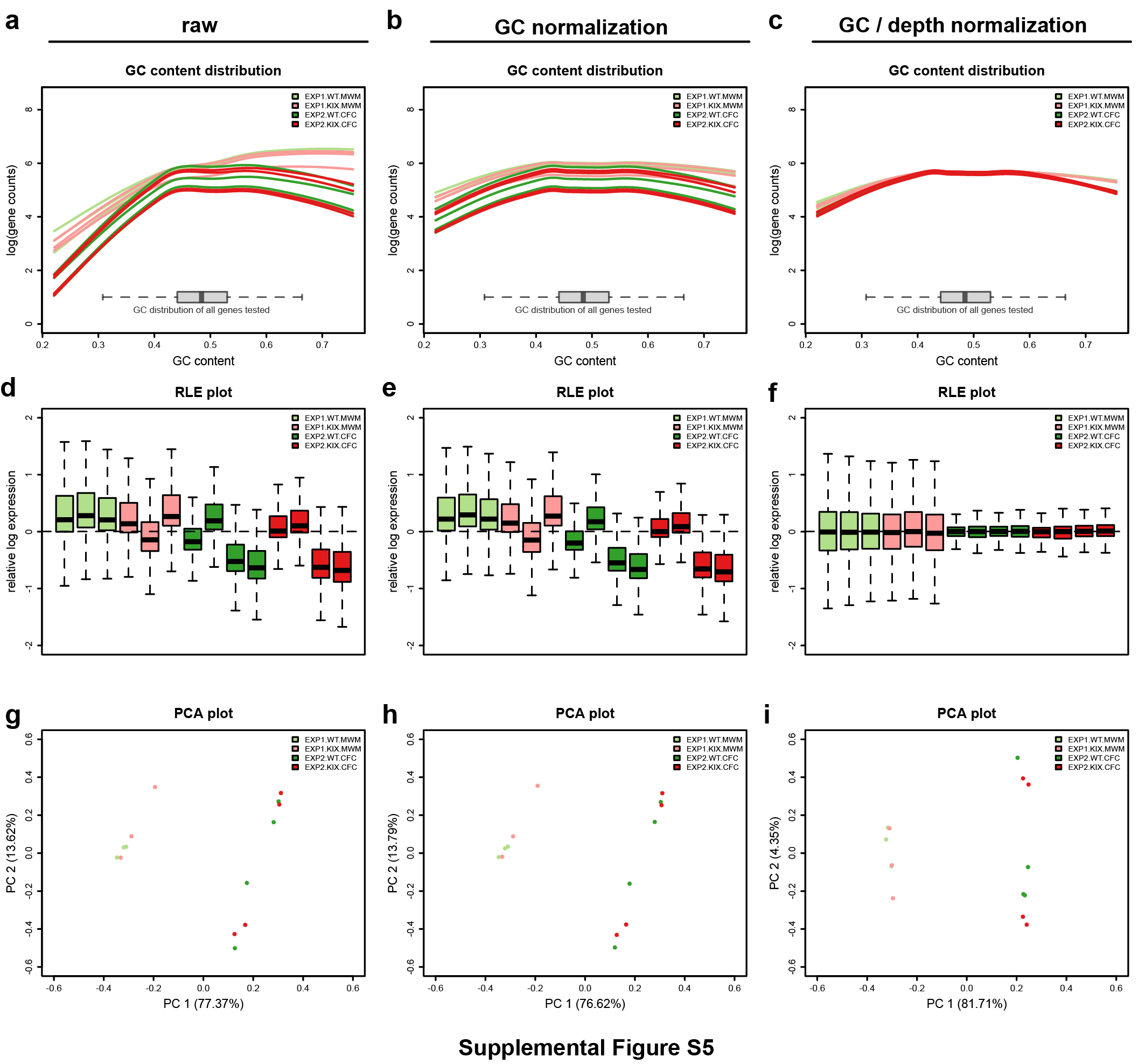
**

**Figure S10.**

**
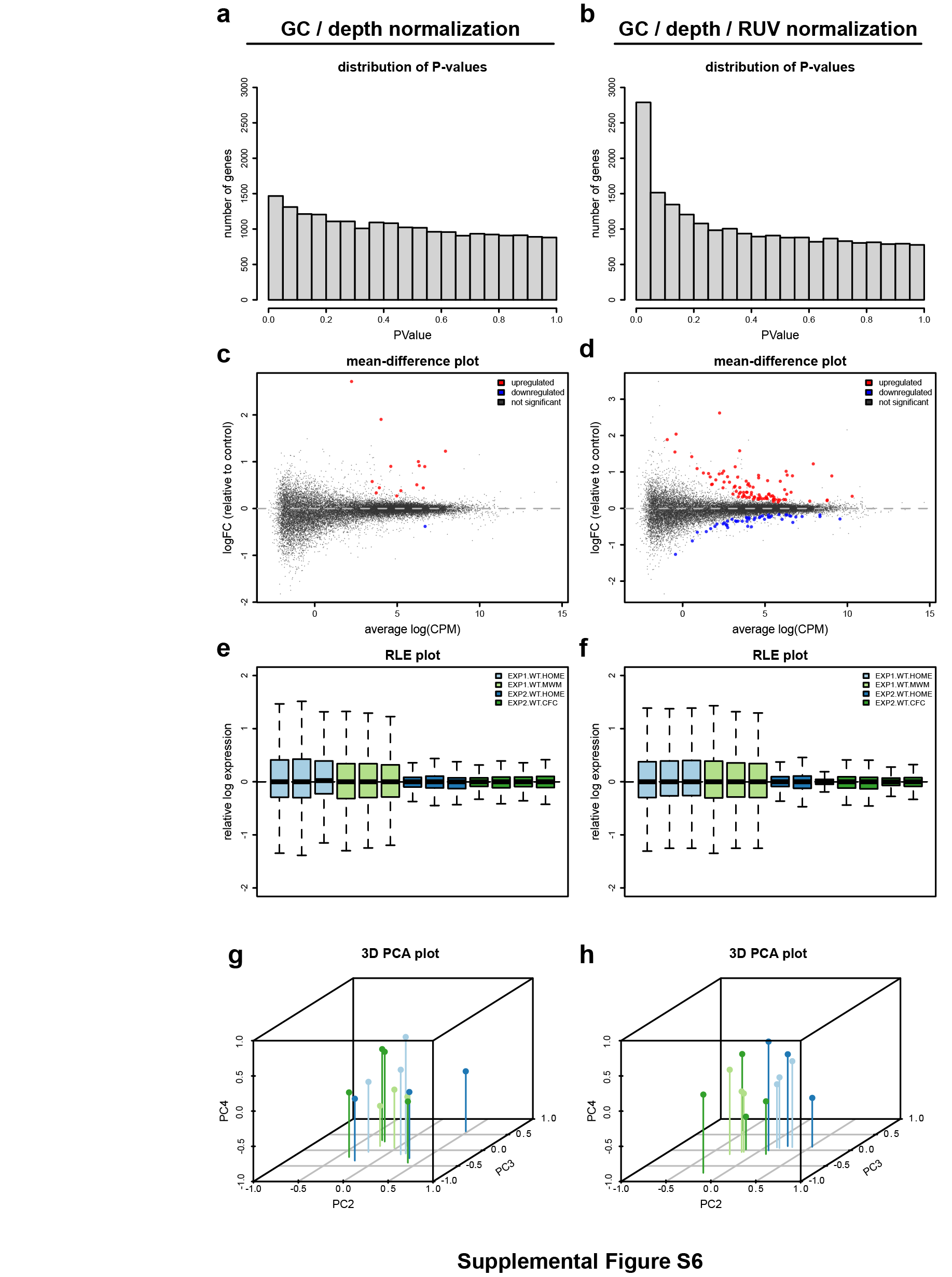
**

**Figure S11.**

**
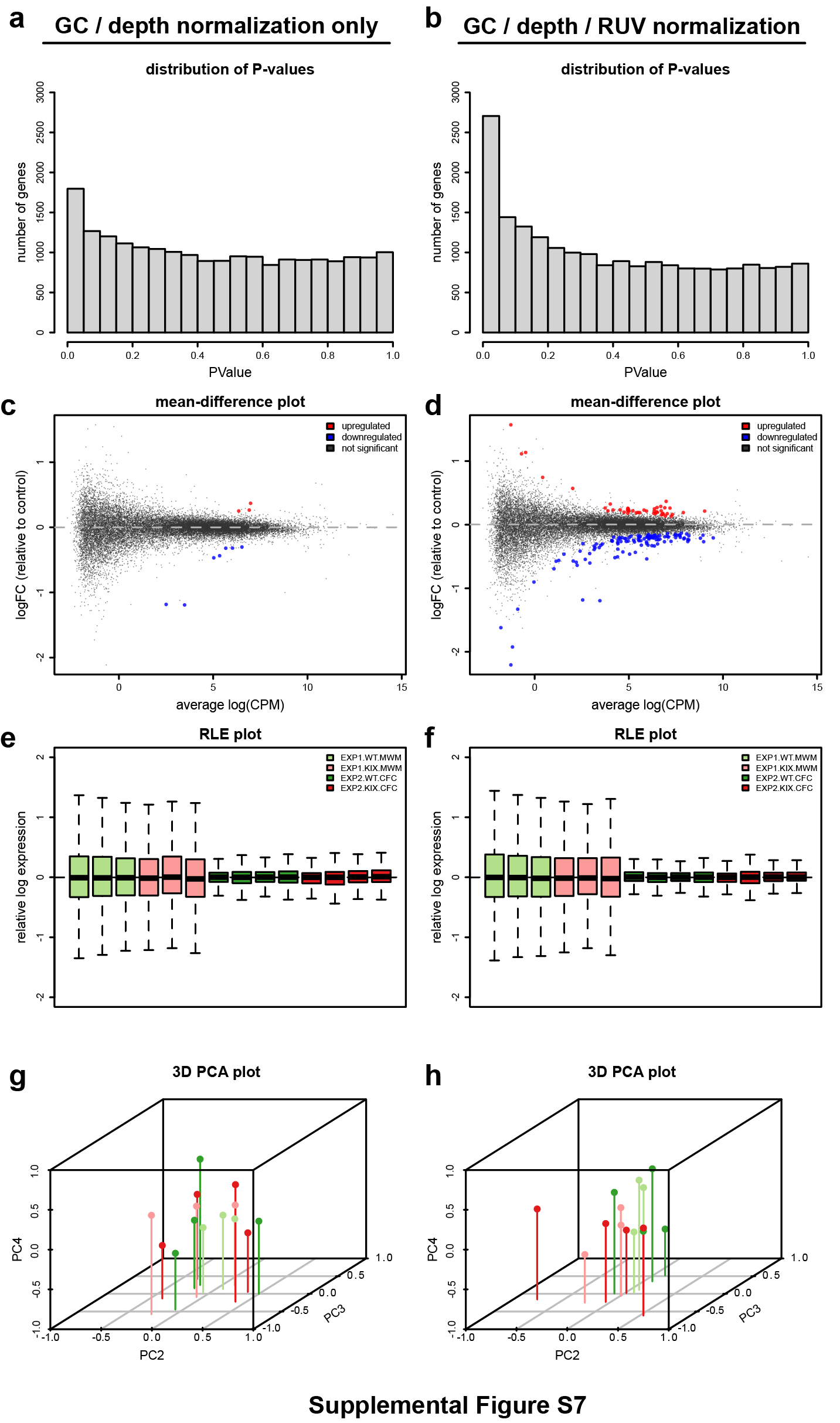
**
